# Melanoma cell line-derived exosomal miR-424-5p: a key promoter of angiogenesis through LATS2 interaction

**DOI:** 10.32604/or.2024.050878

**Published:** 2025-01-16

**Authors:** JUNWEI DU, QIANG ZHANG, JING ZHANG, MAIERDANJIANG MAIHEMUTI, HAIYANG HE, RENBING JIANG

**Affiliations:** Department of Bone and Soft Tissue Tumors and Melanoma, Affiliated Tumor Hospital of Xinjiang Medical University, Urumqi, 830000, China

**Keywords:** Exosomal miR-424-5p, Large tumor suppressor kinase 2 (LATS2), Cell proliferation, Cancer progression, Therapeutic targets

## Abstract

**Objectives:**

Melanoma is a highly aggressive and metastatic form of cancer, and the role of exosomal microRNAs (miRNAs) in its progression remains largely unexplored. This study aimed to investigate the effects of melanoma cell-derived exosomal miR-424-5p on angiogenesis and its underlying mechanisms.

**Methods:**

Exosomes were isolated from melanoma cell lines A375 and A2058, and their effects on the proliferation, migration, and angiogenesis of human umbilical vein endothelial cells (HUVECs) were examined. The interaction between miR-424-5p and its target gene, large tumor suppressor kinase 2 (LATS2), was analyzed using luciferase reporter assays and functional experiments. *In vivo*, tumor growth and angiogenesis were studied in a xenograft model using nude mice.

**Results:**

Melanoma cell-derived exosomes could be internalized by HUVECs, which promoted proliferation, migration, and angiogenesis. miR-424-5p was highly expressed in melanoma cells and their exosomes, and its inhibition in exosomes suppressed HUVEC proliferation, migration, and angiogenesis. LATS2 was identified as a direct target of miR-424-5p, and its silencing reversed the inhibitory effects of miR-424-5p inhibition on HUVEC functions. *In vivo*, exosomes derived from miR-424-5p-inhibited melanoma cells suppressed tumor growth and angiogenesis in xenograft models.

**Conclusions:**

Melanoma cell-derived exosomal miR-424-5p promotes angiogenesis by targeting LATS2, contributing to melanoma progression. Targeting the exosomal miR-424-5p/LATS2 axis could be a potential therapeutic strategy for melanoma.

## Introduction

Melanoma, a malignant tumor originating from melanocytes, is notorious for its high mortality rate, propensity for metastasis, and recurrence [1]. Global statistics from 2020 documented 324,635 newly diagnosed melanoma patients and 57,043 associated deaths, with approximately 20,000 new cases annually in China alone [2,3]. There has been an increasing incidence of melanoma over the past few decades [4]. Despite therapeutic advancements, melanoma’s tendency for distant metastasis, recurrence, and drug resistance continues to pose significant challenges, highlighting the need for a better understanding of its pathogenesis and the development of novel therapeutic strategies [5–7]. Of note, neoangiogenesis is an acknowledged characteristic of tumor development, allowing cancerous cells to cope with metabolic stress via a newly formed vascular source. This process has been identified in melanoma as well, with vascular proliferation linked to heightened aggressiveness and a more unfavorable prognosis [8].

Large tumor suppressor kinase 2 (LATS2) is recognized as a critical regulator in the progression of various cancers, including prostate, pancreatic, and melanoma [9–11]. In prostate cancer, LATS2 has been found to suppress cell proliferation through inhibiting the androgen receptor signaling pathway [10]. Moreover, LATS2 can impede pancreatic cancer cell proliferation via the Hippo signaling pathway, leading to the elimination of F-box protein 22 (FBXO22)-mediated oncogenic effects [12]. Notably, the downregulation of LATS2 in melanoma cells stimulates cell growth, suggesting its potential role as a tumor suppressor in melanoma development [9]. However, the mechanisms underlying LATS2 expression regulation, particularly by non-coding RNAs such as micro RNAs (microRNAs), remain elucidated in melanoma.

Exosomes, small lipid bilayer membrane vesicles ranging in size from 30–150 nm, are constantly produced by cells and can be found in a wide range of body fluids [13]. These vesicles are capable of transporting different substances like proteins, nucleic acids, and lipids, which play crucial roles in the development, progression, and spread of cancer [14]. As a result, exosomes have emerged as important biomarkers for the early detection of cancer [15]. Moreover, microRNAs (miRNAs), short non-coding RNA molecules typically made up of 20–25 bases, bind to the 3′-untranslated region (3′-UTR) of their target messenger RNAs (mRNAs), leading to the degradation of the mRNA or blocking its translation. This process effectively inhibits the post-transcriptional expression of genes in a variety of cells [16]. The presence of miRNAs has been closely associated with key cellular functions like proliferation, migration, and angiogenesis in a range of tumor cells [17,18].

MiR-424-5p has been identified as a key player in the initiation and progression of various types of cancers, including nasopharyngeal carcinoma, skin squamous cell carcinoma, and cholangiocarcinoma [19–21]. In nasopharyngeal carcinoma, miR-424-5p facilitates cell growth, movement, and metastasis by targeting LATS2, a gene that suppresses tumor progression [21]. Similarly, in skin squamous cell carcinoma, miR-424-5p enhances cancer stem cell properties and tumorigenicity by downregulating the negative regulators of the Notch signaling pathway [10]. This miRNA can also regulate diverse cellular processes in other cancers, such as suppressing ectopic endometrial stromal cell proliferation, invasion, and migration by targeting CREB1 [22] and impeding the growth of breast cancer cells [23]. Nevertheless, the role of exosome-derived miR-424-5p in melanoma, especially in tumor-associated angiogenesis, remains largely unexplored.

The present study aimed to investigate the functional effects and underlying mechanisms of melanoma cell-derived exosomal miR-424-5p on angiogenesis, a critical process in tumor progression. Exosomes were isolated from melanoma cell lines A375 and A2058, and their effects on the proliferation, migration, and angiogenesis of human umbilical vein endothelial cells (HUVECs) were examined *in vitro*. The interaction between miR-424-5p and its putative target LATS2 was investigated through luciferase assays and functional experiments. Additionally, an *in vivo* xenograft model was employed to assess the impact of exosomal miR-424-5p on tumor growth and angiogenesis. Our findings suggest that targeting the exosomal miR-424-5p/LATS2 axis could represent a potential therapeutic strategy for melanoma.

## Materials and Methods

### Cell cultivation

Melanocytes from normal human skin (PIG1), melanoma cell lines (A375 and A2058), as well as human umbilical vein endothelial cells (HUVECs), were obtained from the Shanghai Cell Bank of the Chinese Academy of Sciences (Shanghai, China). The cells were seeded at a density of 1 × 10^5^ cells/mL in cell culture flasks and maintained in RPMI1640 medium (Gibco, Waltham, MA, USA) with 10% fetal bovine serum at 5% CO_2_ and 37°C. Scramble si-RNA (si-NC) and LATS2 targeting si-RNA (si-LATS2) were purchased from MedChemExpress (catalog# 4392421, Shanghai, China). miRNA inhibitor (catalog: miR311720164508-4-5) and mimic (catalog: miR10001341-1-5) were synthesized by RiboBio (Guangzhou, China). The transfection of these molecules (100 nM) were conducted using using Lipofectamine 2000 (catalog #11668, Invitrogen, Carlsbad, CA, USA).

### Isolation of exosomes

A total 15 mL cell culture supernatant from 5 × 10^6^ cultured A375 or A2058 cells was placed in a tube for centrifugation at 100,000 g for 15 min at 4°C, followed by the reconstitution with 1 mL phosphate-buffered saline (PBS) (Catalog#20012019, Gibco, Waltham, MA, USA) The sample was then subjected to centrifugation at 310 g at 4°C for 10 min to remove cell debris. Subsequently, the supernatant underwent another round of centrifugation at 4°C for 10 min and the liquid phase was then placed into a ultracentrifuge tube (Beckman Coulter, Brea, CA, USA) for 30-min centrifugation at 10,000 g at 4°C. In a similar fashion, the supernatant was transferred into the ultracentrifuge tube and centrifuged for 70 min at 100,000 g at 4°C. The resulting clear precipitate was then resuspended in 250 μL of PBS, followed by a 30-min centrifugation at 10,000 g to collect the supernatant. After another 70-min centrifugation at 100,000 g and 4°C, the transparent precipitate was resuspended in 250 μL of PBS to yield the exosome solution, which was divided into aliquots and stored at −80°C for future experiments.

### Morphological analysis of exosomes via transmission electron microscopy

A 50 μL aliquot of the exosome sample was added onto a 200-mesh copper grid and allowed to stand at room temperature for 5 min. Excess fluid was carefully removed with filter paper. The sample was then negatively stained with 1% phosphotungstic acid (Solarbio, Beijing, China) for 1 min, followed by rinsing 2 times with distilled water. Excess liquid was again removed using filter paper, and the sample was allowed to dry. The exosome morphology was then examined using a JEM-1400plus transmission electron microscope (JEOL, Tokyo, Japan) at an acceleration voltage of 80 kV.

### Nanoparticle tracking analysis (NTA) for determining exosome concentration and size

Exosome samples were diluted to a concentration of 3 × 10^7^ to 5 × 10^7^ particles/mL using 1 × PBS. The diluted samples were then analyzed using a ZetaView PMX110 instrument (Particle Metrix, Meerbusch, Germany). The resulting data were processed using ZetaView 8.04.02 software (Particle Metrix, Meerbusch, Germany) to determine the exosome concentration and size distribution.

### Protein analysis via western blot

Radioimmunoprecipitation Assay (RIPA) lysis buffer (containing 1% Phenylmethylsulfonyl Fluoride (PMSF), Servicebio, Wuhan, China) was used to extract cellular proteins from various cell groups, which were then collected via low-speed centrifugation. A Bicinchoninic Acid (BCA) protein assay kit (Beyotime, Nantong, China) was employed to measure the total protein level. Sodium Dodecyl Sulfate-Polyacrylamide Gel Electrophoresis (SDS-PAGE) electrophoresis was conducted to separate the protein samples using a 5% stacking gel and a 10% resolving gel (Servicebio, Wuhan, China), with 20 μg of protein samples loaded per lane. For protein transfer onto Polyvinylidene Difluoride (PVDF) membranes (Millipore, Burlington, MA, USA), electroblotting was conducted for 90 min at a current of 200 mA. The membranes were blocked for 2 h using a 10% non-fat milk solution (Servicebio, Wuhan, China), and later incubated with primary antibodies at 4°C overnight. After rinsing three times with Tris-Buffered Saline with Tween 20 (TBST) for 15 min each, corresponding secondary antibodies were introduced to incubate the membranes for 2 h at room temperature. Following three additional TBST washes, proteins were visualized using an Enhanced Chemiluminescence (ECL) chemiluminescent solution (Servicebio, Wuhan, China). The resulting western blot images were analyzed using ImageJ software (National Institutes of Health, Bethesda, MD, USA). The antibodies utilized were as follows: CD63 (Cell Signaling Technology, Danvers, MA, USA, #52090); CD9 (Cell Signaling Technology, Danvers, MA, USA, #98327); TSG101 (Cell Signaling Technology, Danvers, MA, USA, #72312); LATS2 (Cell Signaling Technology, Danvers, MA, USA, #5888); Glyceraldehyde 3-Phosphate Dehydrogenase (GAPDH) (Cell Signaling Technology, Danvers, MA, USA, #92310); Horseradish Peroxidase (HRP)-conjugated anti-rabbit and anti-mouse IgG (Cell Signaling Technology, Danvers, MA, USA, #7074, #7076).

### Immunofluorescence staining

The internalization of Paul Karl Horan 67 (PKH67)-labeled exosomes from Exo-A375 (exosomes from A375 cells) and Exo-A2058 (exosomes from A2058 cells) by HUVEC cells was assessed using immunofluorescence analysis. After fixation with 4% paraformaldehyde for 20 min, cells were permeabilized with 0.1% Triton X-100 for 10 min, and then blocked with 3% Bovine Serum Albumin (BSA) for 30 min, followed by overnight incubation at 4°C with anti-PKH67 antibody (Abcam, Cambridge, UK, ab204951). Subsequently, cells were incubated for 1 h at room temperature with Cyanine3 (Cy3)-conjugated goat anti-rat secondary antibody (Abcam, Cambridge, UK, ab6953). 4′,6-diamidino-2-phenylindole (DAPI) (Servicebio, Wuhan, Hubei, China) was used for nuclear staining, and images were captured for analysis by laser confocal microscope (FV3000, Olympus, Tokyo, Japan).

### Cell proliferation assessment via cell count kit (CCK)-8 assay

To measure cell proliferation, the CCK-8 assay was utilized. HUVEC cells were co-cultured with Exo-A375 and Exo-A2058 exosomes for varying durations of 24, 48, and 72 h. Following this, the treated cells (5 × 10^3^ cells/well) were seeded into 96-well plates and mixed with 10 μL of CCK-8 solution (catalog#C0065FT, Beyotime, Nantong, China). After an hour of incubation at 37°C, the absorbance at 450 nm (OD450) was recorded using a spectrophotometer (Bio-Rad, Hercules, CA, USA).

### Transwell migration assay

A Transwell chamber (8.0 μm pore size, provided by Corning from NY, USA) was used for the migration assay. A cell suspension containing 1 × 10^5^ cells was prepared and 500 μL of this suspension was inoculated in the top chamber, with an additional 600 μL of complete medium in the bottom chamber. The setup was then placed in an incubator at 37°C with 5% CO_2_ for 24 h. Following fixation using 4% paraformaldehyde, the cells were stained using crystal violet (catalog#C0121 Beyotime, Nantong, China). The cells that migrated through the filter membrane into the bottom chamber were observed and counted using a microscope (Olympus, Tokyo, Japan) at a 200× magnification.

### Tube formation assay

The Matrigel matrix gel (catalog#356231, Corning, NY, USA) was thawed overnight at 4°C. 24-well plates and pipette tips were pre-chilled at −20°C. The matrix gel was spread evenly across the pre-chilled 24-well plate using the cooled pipette tips, ensuring no air bubbles were present. 200 μL of gel was added to each well, with three replicate wells for each group. Plates were placed at 4°C to allow for even distribution of the gel, followed by transfer to an incubator for gel solidification after 30 min. Cells were harvested and adjusted to a density of 1 × 10^5^ cells/mL, and 1 mL of cell suspension was added to the corresponding well in the 24-well plates before returning to the incubator. Tube formation was observed 24 h later using an inverted microscope (Olympus X51, Tokyo, Japan), and images were documented.

### Quantitative real-time PCR (qRT-PCR)

Cells under various treatments were collected, and TRIzol reagent (*catalog* #15596026, Invitrogen, Carlsbad, CA, USA) was added to extract total cellular RNA from 1 × 10^6^ cells. Meanwhile, a Thermo NanoDrop 2000 (catalog # ND-2000 Thermo Fisher Scientific, Waltham, MA, USA) was utilized to determine RNA content and quality. For miRNA quantification, complementary DNA was prepared from 1 μg RNA sample through reverse transcription using miRNA PrimeScript RT kit (Catalog#638315 Takara, Shiga, Japan). For mRNA determination, PrimeScript™ RT kit with gDNA Eraser (Takara, Shiga, Japan) was utilized to synthesize complementary DNA from 1 μg RNA sample. SYBR Premix Ex Taq TM II (Cat#DRR820A Takara, Shiga, Japan) was utilized to quantify complementary DNA sample through a real-time qRT-PCR assay carried out using a Bio-Rad CFX96 system (Bio-Rad, Hercules, CA, USA). The 2^−ΔΔCT^ method was utilized to determine gene expression levels. The primers utilized for the qRT-PCR were as follows: miR-424-5p (primer for miRNA analysis): F: AGCAGCAATTCATGTTTTG, R: GAACATGTCTGCGTATCTC; U6 (primer for miRNA analysis) F: GCTTCGGCAGCACATATACTAAAAT, R: CGCTTCACGAATTTGCGTGTCAT, LATS2 (primer for mRNA): F: ACTTTTCCTGCCACGACTTATTC, R: GATGGCTGTTTTAACCCCTCA; GAPDH (primer for mRNA): F: GGGAAACTGTGGCGTGAT, R: GAGTGGGTGTCGCTGTTGA.

### Dual-luciferase reporter assay

To conduct the experiment, fragments of the LATS2 mRNA 3′-UTR with either a normal or altered miR-424-5p binding region. These fragments were inserted into the pmirGLO vector from (Cat#E1330 Promega, Madison, WI, USA). generating wild-type (WT) LATS2 3′-UTR or mutant (MUT) LATS2 3′-UTR constructs. These luciferase reporter plasmids were later transfected into HUVECs along with miR-424-5p or miR-NC using Lipofectamine 2000 (Invitrogen, Carlsbad, CA, USA). After transfection for 48 h, the Dual-Luciferase Assay Kit (Catalog#E1910 Promega, Madison, WI, USA) was employed to measure luciferase activities.

### In vivo tumor formation in nude mice

An *in vivo* tumorigenic model was established using 8-week old female nude mice (Cyagen, Guangzhou, China). Mice were housed in a specific-pathogen-free environment under controlled conditions of temperature (22 ± 2°C) and humidity (50 ± 10%), with a 12-hour light/dark cycle. Mice (3–5 per cage) were housed in groups in individually ventilated cages with access to standard rodent chow and filtered water. Animal health was monitored daily by trained personnel. Logarithmic-phase A375 melanoma cells were collected and their concentration was adjusted to 2 × 10^7^ cells/mL. Later, cell suspension aliquots (0.1 mL/per animal) were given into each mouse through subcutaneous injection. Tumor size (mm^3^) was measured weekly using the formula: (length × width^2^)/2. When subcutaneous tumors reached 200 mm^3^, the mice were randomly assigned into 3 groups (n = 6 in each group). The control group was administrated with PBS via subcutaneous injection. The intervention groups were administrated with Exo-A375+NC inhibitor or Exo-A375+miR-424-5p inhibitor via the tail vein (twice weekly). After three weeks, euthanasia was performed using sodium pentobarbital (catalog#1507002; Sigma-Aldrich, St. Louis, Missouri, USA; Merck KGaA 100 mg/kg), followed by cervical dislocation. The tumors were excised for subsequent analysis. Each animal experimental procedure gained approval from Animal Ethics Committee of Affiliated Tumor Hospital of Xinjiang Medical University (approval number: 2023-DL154). The experimental protocol was performed in accordance with the relevant guidelines and regulations of the Basel Declaration. The study is reported in accordance with ARRIVE guidelines (https://arriveguidelines.org, accessed on 20 January, 2023).

### Immunohistochemical (IHC) analysis

Xenograft tumors were subjected to paraffin embedding and sectioning into 4-μm slices, followed by 1-h incubation with anti-CD34 primary antibody (Cell Signaling Technology, Danvers, MA, USA, cat#3569) and goat anti-mouse secondary antibodies at room temperature. Following DAB staining and hematoxylin counterstaining (catalog#C0105 Beyotime, Nantong, China), the fluorescence microscope (Olympus X51, Tokyo, Japan) was utilized to capture images.

### Statistical evaluation

Results from a minimum of three separate assays were represented as mean ± standard deviation. Data were analyzed using GraphPad Prism 7 software (GraphPad Software, San Diego, CA, USA). One-way ANOVA or Student’s *t*-test was applied to evaluate differences between groups, with *p* < 0.05 indicating statistical significance.

## Results

### The proliferation, migration, and angiogenesis of HUVECs are enhanced by melanoma cell-derived exosomes

**Figure 1:**
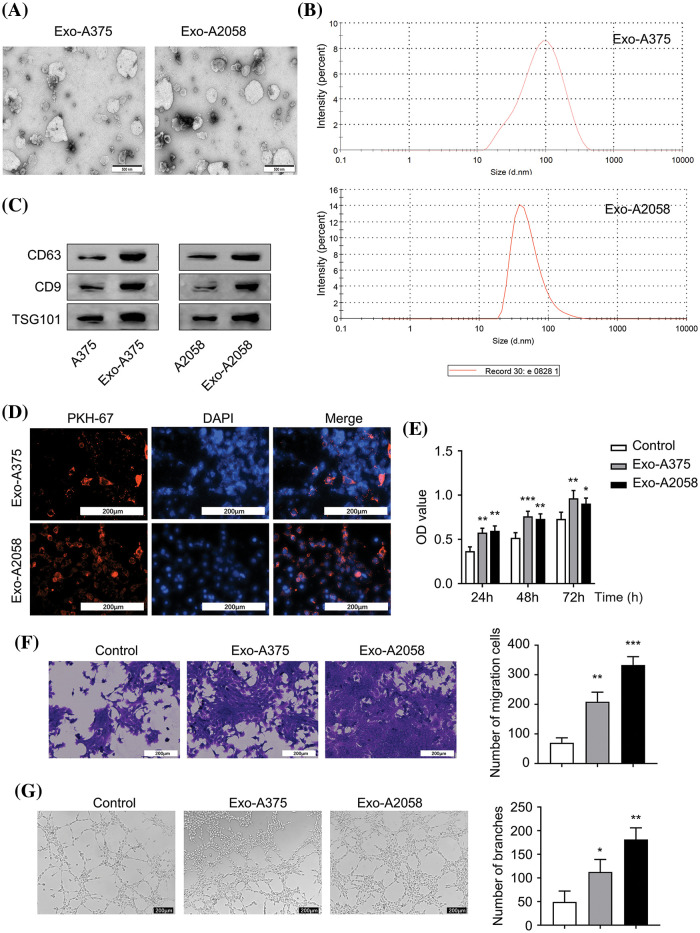
Melanoma cell-derived exosomes promote proliferation, migration and angiogenesis of HUVEC cells. (A): Exosomes were visualized using electron microscopy. (B): Nanoparticle Tracking Analysis (NTA) was conducted for determining exosome size originating from A375 and A2058 cells. (C): Exosomal markers (CD63, CD9, and TSG101) levels in A375 and Exo-A375 exosomes, as well as in A2058 cells and Exo-A2058 exosomes, was assessed using Western blot analysis. (D): The internalization of PKH67-labeled Exo-A375 and Exo-A2058 exosomes in HUVEC cells was detected through immunofluorescence analysis. (E): CCK-8 assay was utilized for evaluating proliferation levels of HUVEC cells co-cultured with different groups (control, Exo-A375, and Exo-A2058) throughout 24, 48, and 72 h. (F): A Transwell assay was conducted for measuring HUVEC migration after co-culture with different groups (control, Exo-A375, and Exo-A2058) after 24 h. (G): Tube formation assay in different groups of HUVECs (control, Exo-A375, and Exo-A2058). Every assay was carried out thrice. *p < 0.05, **p < 0.01, ***p < 0.001, *vs*. control group.

Exosomes (Exo-A375 and Exo-A2058) extracted from A375 and A2058 cells exhibited typical exosomal vesicles with varying sizes (Fig. 1A). NTA analysis revealed that the peak sizes of Exo-A375 ranged from 50–100 nm, while those of Exo-A2058 ranged from 50–150 nm (Fig. 1B). These indicate that the size of exosomes from different cell lines could be different. Next, we detect different exosomal markers (CD63, CD9, and TSG101) in the cell and exosomal samples. We showed that Exo-A375 and Exo-A2058 samples showed an elevated enrichment of these exosomal markers (Fig. 1C). Tracking of exosomes showed that PKH-67-labeled Exo-A375 and Exo-A2058 were efficiently internalized by HUVEC cells (Fig. 1D). The overall proliferation of HUVEC in Exo-A375 and Exo-A2058 groups remarkably increased at 24, 48, and 72 h in comparison with the control group (Fig. 1E, *p* < 0.05). HUVEC migration of Exo-A375 and Exo-A2058 groups dramatically increased relative to the control group (Fig. 1F, *p* < 0.01). Similarly, tube-forming ability in HUVEC cells was significantly enhanced in the Exo-A375 and Exo-A2058 groups in comparison with the control group (Fig. 1G, *p* < 0.01). Therefore, exosomes derived from melanoma cells enhance HUVEC proliferation, migration, and angiogenesis.

### Suppression of HUVEC proliferation, migration, and angiogenesis via miR-424-5p knockdown within melanoma cell-derived exosomes

**Figure 2:**
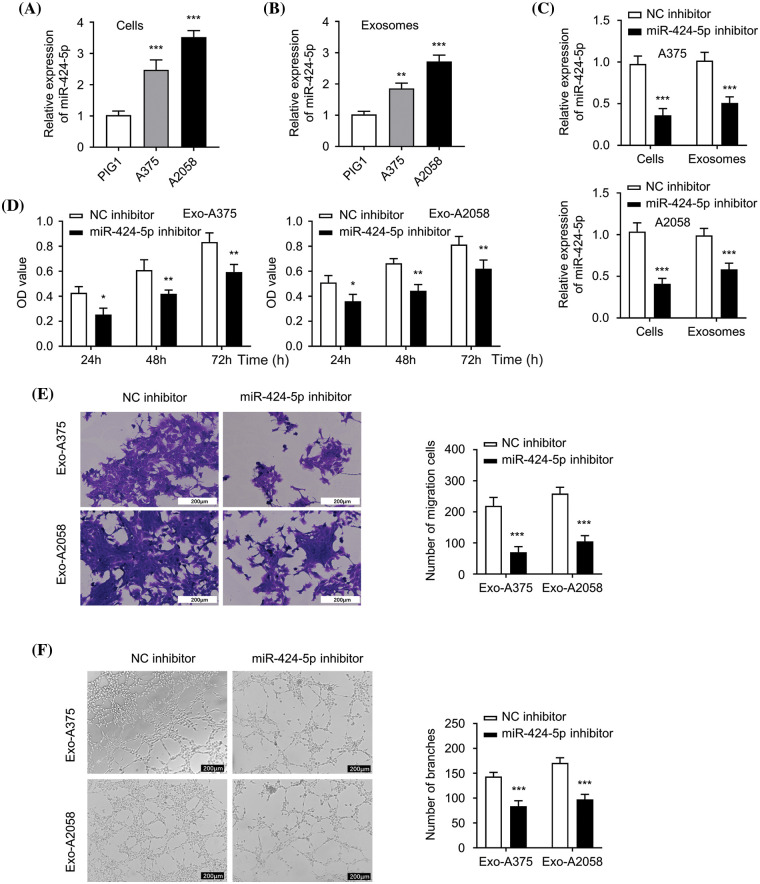
Exosome derived from melanoma cells with miR-424-5p knockdown suppresses HUVEC proliferation, migration and angiogenesis. (A): miR-424-5p expression levels within healthy human epidermal melanocytes and melanoma cells (A375 and A2058) were quantified using qRT-PCR. (B): miR-424-5p expression within exosomes (Exo-miR-424-5p) derived from normal human epidermal melanocytes and in exosomes (Exo-A375 and Exo-A2058) derived from melanoma cell lines A375 and A2058 were measured using qRT-PCR. (C): A375 and A2058 cells were transfected into NC inhibitor and miR-424-5p inhibitor, then miR-424-5p level within these cells and exosomes derived from these cells was assessed using qRT-PCR. (D): HUVEC cell proliferation after co-culture with different groups of exosomes as indicated for 24/48/72 h was determined using the CCK-8 assay. (E): Migration level of each group of cells was evaluated by a Transwell assay. (F): The tube formation level of each group of cells was assessed by a tube formation assay. Every assay was completed thrice. *p < 0.05, **p < 0.01, ***p < 0.001, *vs*. NC inhibitor group.

The level of miR-424-5p significantly increased in A375 and A2058 cells compared to PIG1 cells (Fig. 2A, *p* < 0.001). Likewise, the miR-424-5p levels in the Exo-A375 and Exo-A2058 showed a marked increase compared to the exosomal sample from PIG1 cells (Fig. 2B, *p* < 0.01). In contrast, miR-424-5p levels significantly decreased in A375 and A2058 cells in the miR-424-5p inhibitor group, as well as in the Exo-A375 and Exo-A2058 group with miR-424-5p transfection (Fig. 2C, *p* < 0.001). HUVEC cell proliferation declined significantly after co-culture with Exo-A375 and Exo-A2058 from the miR-424-5p inhibitor group at 24/48/72 h compared to the NC inhibitor group (Fig. 2D, *p* < 0.05). The migratory and tube-forming capacities of HUVECs under treatment with Exo-A375 and Exo-A2058 from the miR-424-5p inhibitor group significantly decreased compared to the NC inhibitor group (Fig. 2E,F, *p* < 0.001). These findings suggest that miR-424-5p knockdown in melanoma cell-derived exosomes suppresses HUVEC proliferation, migration, and angiogenesis.

### LATS2 serves as miR-424-5p’s target

**Figure 3:**
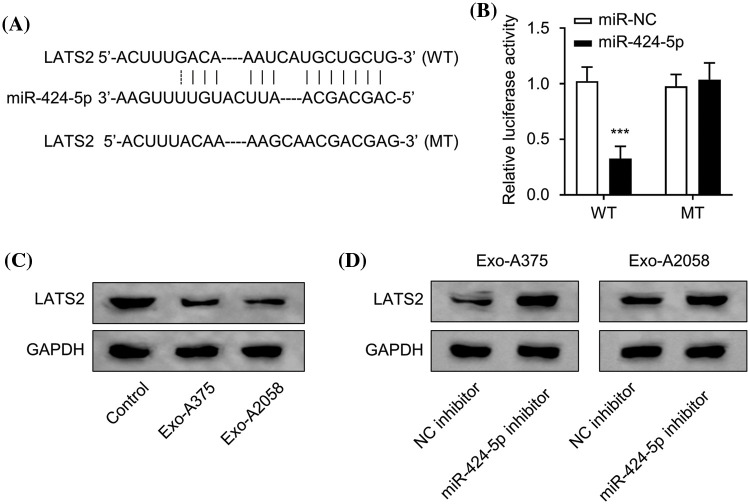
miR-424-5p targets LATS2. (A): Starbase prediction indicates that miR-424-5p targets LATS2 sites. (B): The luciferase reporter gene assay was conducted for confirming miR-424-5p binding to *LATS2* 3′-UTR. (C): Western-blotting analysis was performed for assessing LATS2 protein expression in HUVEC cells co-cultured with different groups (control, Exo-A375, and Exo-A2058) for 24 h. (D): Western-blotting assay was also conducted for measuring LATS2 protein level in HUVEC cells co-cultured with Exo-A375 and Exo-A2058 for 24 h. Every assay was carried out thrice. ***p < 0.001, *vs*. miR-NC group.

Next, we utilized Starbase prediction analysis (http://starbase.sysu.edu.cn/, accessed on 16 October, 2022) to predict the mRNA target of miR-424-5p, which is based on various computational algorithms for predicting miRNA target prediction based on sequence complementarity. miR-424-5p was found to target the 3′UTR sites of LATS2 mRNA (mRNA accession number: NM_014572) (Fig. 3A). The wild-type (WT) or mutated (MUT) sequences of LAST2 mRNA 3′UTR were cloned into pmirGLO miRNA reporter for interaction analysis. According to the luciferase reporter gene assay, miR-424-5p overexpression remarkably suppressed the luciferase activity of the WT reporter, the effect was abolished after mutation of the predicted *LATS2* 3′-UTR binding sites in the MUT reporter (Fig. 3B, *p* < 0.001). Compared with the control group, LATS2 protein levels within HUVEC cells evidently decreased following treatment with Exo-A375 or Exo-A2058 (Fig. 3C). Furthermore, in HUVECs co-cultured with Exo-A375 or Exo-A2058 exosomes, the transfection of miR-424-5p inhibitor could increase the LATS2 protein level in comparison with the NC inhibitor (Fig. 3D). The above findings demonstrate LATS2 as miR-424-5p’s target.

### Regulation of HUVEC proliferation, migration, and angiogenesis through exosome-derived miR-424-5p via targeting LATS2

**Figure 4:**
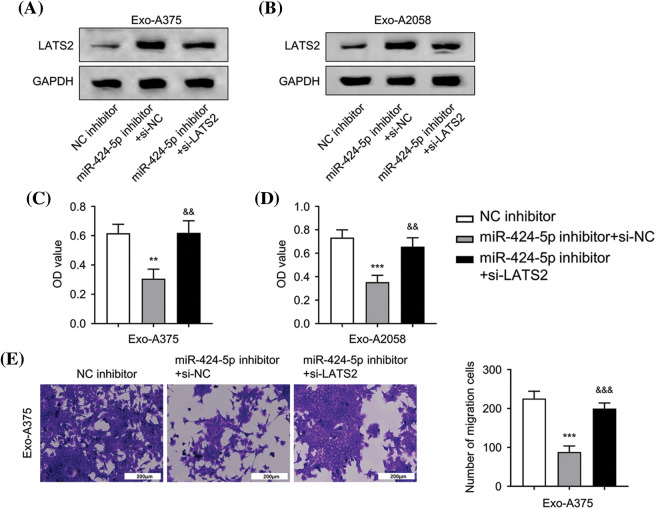

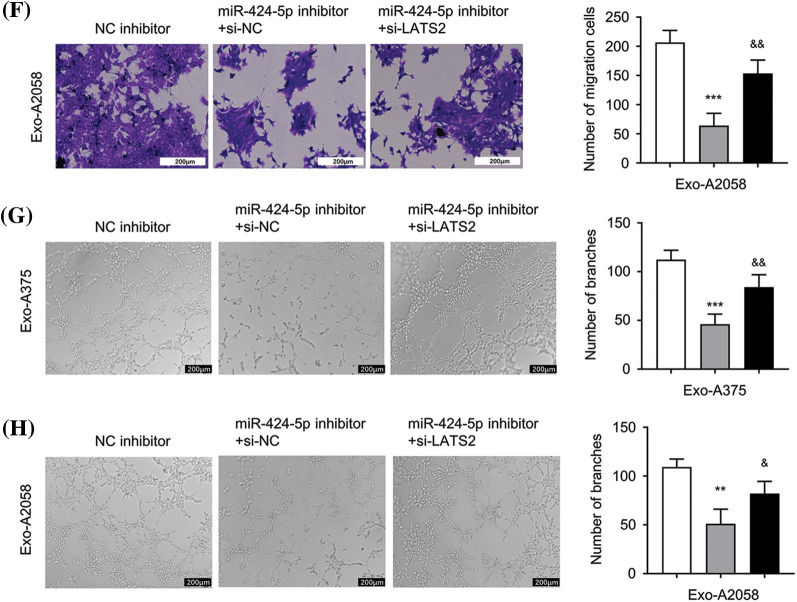
Exosome-derived miR-424-5p regulates HUVEC proliferation, migration and angiogenesis of HUVEC cells via LATS2. A: Western-blotting analysis was conducted for measuring LATS2 protein levels in different groups of HUVECs cultivated with Exo-A375 (NC inhibitor, miR-424-5p inhibitor+si-NC, and miR-424-5p inhibitor+si-LATS2) after 24 h. B: Western-blotting assay was also conducted for assessing LATS2 protein expression within different groups of HUVECs cultivated with Exo-A2058 (NC inhibitor, miR-424-5p inhibitor+si-NC, and miR-424-5p inhibitor+si-LATS2) after 24 h. C: CCK-8 assay was carried out for evaluating HUVEC proliferation in different groups of HUVECs cultivated with Exo-A375 (NC inhibitor, miR-424-5p inhibitor+si-NC, and miR-424-5p inhibitor+si-LATS2). D: CCK-8 assay was also carried out for measuring HUVEC proliferation in different groups cultivated with Exo-A2058 (NC inhibitor, miR-424-5p inhibitor+si-NC, and miR-424-5p inhibitor+si-LATS2). E: The Transwell assay was conducted for assessing HUVECs cultivated with Exo-A375 (NC inhibitor, miR-424-5p inhibitor+si-NC, and miR-424-5p inhibitor+si-LATS2). F: The Transwell assay was conducted for assessing HUVECs cultivated with Exo-A2058 (NC inhibitor, miR-424-5p inhibitor+si-NC, and miR-424-5p inhibitor+si-LATS2). G: The tube formation assay was performed for evaluating tube-forming ability in HUVECs cultivated with Exo-A375 (NC inhibitor, miR-424-5p inhibitor+si-NC, and miR-424-5p inhibitor+si-LATS2). H: The tube formation assay was performed for evaluating tube-forming ability in HUVECs cultivated with Exo-A2058 (NC inhibitor, miR-424-5p inhibitor+si-NC, and miR-424-5p inhibitor+si-LATS2). Every assay was carried out thrice. **p < 0.05, ***p < 0.001, *vs*. NC inhibitor group; ^&^*p* < 0.05, ^&&^*p* < 0.01, ^&&&^*p* < 0.001, *vs*. miR-424-5p inhibitor+si-NC group.

Next, we applied a siRNA targeting LATS2 (si-LATS2) to study its involvement in regulating HUVEC function. WB results demonstrated that silencing LATS2 with si-LATS2 suppressed LATS2 protein upregulation after miR-424-5p inhibitor treatment in HUVECs cultivated with Exo-A375 and Exo-A2058 (Fig. 4A,B). Furthermore, silencing LATS2 could rescue the proliferation, migration, and tube formation capacities in the miR-424-5p inhibitor treatment group (Fig. 4C–H). According to the above findings, LATS2 serves as a downstream mediator of miR-424-5p to modulate the cell proliferation, migration, and angiogenesis of HUVECs.

### In vivo, tumor formation of melanoma cells and angiogenesis are promoted by melanoma cell-derived exosomes

**Figure 5:**
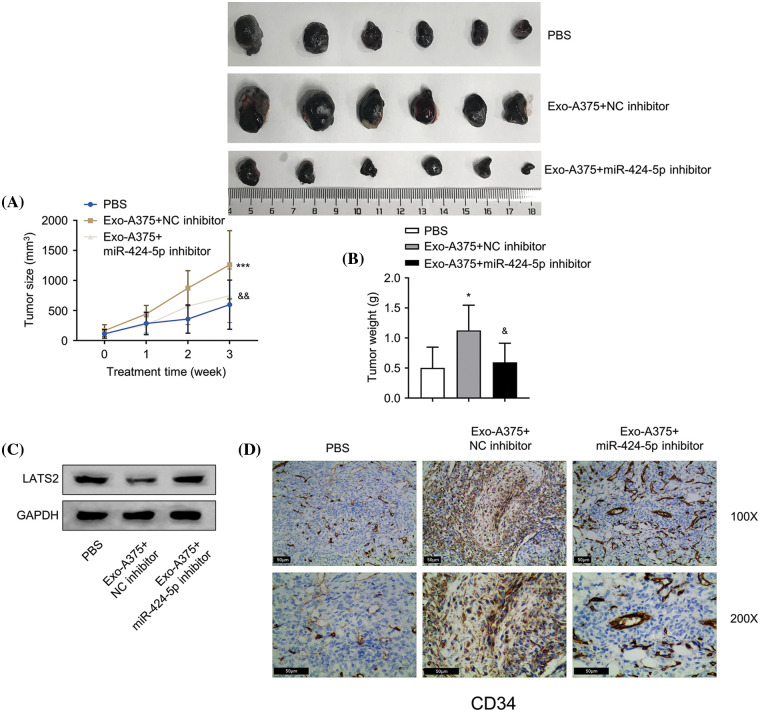
Melanoma cell-derived exosomes promote melanoma cell proliferation and angiogenesis *in vivo*. (A and B): A subcutaneous tumor model was established with A375 cells, then tumor volume and mass were measured. (C): Western-blotting analysis was conducted for detecting LATS2 protein expression in subcutaneous tumors of each group. (D): Immunohistochemistry (IHC) was carried out for detecting CD34 protein level within subcutaneous tumors in different groups. N = 6 in each group. *p < 0.05, ***p < 0.001, *vs*. PBS group; ^&^*p* < 0.05, ^&&^*p* < 0.01, *vs*. Exo-A375-NC inhibitor group.

Next, we sought to examine the influence of Exo-A375 on tumor formation and angiogenesis in nude mice. A375 melanoma cells were inoculated into each mouse through subcutaneous injection until tumor size reached 200 mm^3^. The control group was administrated with PBS via subcutaneous injection. The intervention groups were administrated with Exo-A375+NC inhibitor or Exo-A375+miR-424-5p inhibitor. Exo-A375 administration markedly increased subcutaneous tumor mass and volume in comparison with the PBS group (*p* < 0.001). However, these parameters were partially reduced within the Exo-A375-miR-424-5p inhibitor group (Fig. 5A,B, *p* < 0.001). Concurrently, LATS2 expression in the tumor tissues of the Exo-A375-NC inhibitor group decreased relative to the PBS control group, while the co-administration of miR-424-5p inhibitor increased LATS2 levels (Fig. 5C). Furthermore, Exo-A375 treatment also increased CD34 expression (angiogenic marker) in the tumor tissues, and this effect was suppressed by miR-424-5p inhibitor (Fig. 5D). Collectively, exosomes derived from melanoma cells promote the *in vivo* growth of melanoma cells and angiogenesis within subcutaneous graft tumors.

## Discussion

This study demonstrated that melanoma cell-derived exosomal miR-424-5p promotes angiogenesis, a crucial process for tumor progression. Exosomes from melanoma cell lines A375 and A2058 were internalized by HUVECs, enhancing their proliferation, migration, and angiogenic potential. miR-424-5p was highly expressed in melanoma cell exosomes, and its inhibition suppressed HUVEC functions. LATS2 was identified as a direct target of miR-424-5p, and its silencing reversed the effects of miR-424-5p inhibition. *In vivo*, experiments confirmed that exosomes from miR-424-5p-inhibited melanoma cells suppressed tumor growth and angiogenesis. These findings suggest targeting the exosomal miR-424-5p/LATS2 axis as a potential therapeutic strategy for melanoma.

Tumor-derived exosomes have emerged as crucial mediators of cancer progression, facilitating intercellular communication and modulating the tumor microenvironment [24]. These exosomes can transport various bioactive molecules, including proteins, lipids, and nucleic acids, enabling them to influence recipient cells and promote tumor growth, metastasis, and angiogenesis [25]. For instance, in ovarian cancer, exosomes derived from cancer-associated adipocytes have been shown to promote tumor angiogenesis and metastasis by transferring miR-199a-5p, which targets the Von Hippel-Lindau (VHL) and Notch1 genes [26]. Similarly, in lung cancer, exosomes derived from tumor cells can deliver miR-23a to endothelial cells, enhancing angiogenesis and tumor growth [27]. Our findings align with these studies, demonstrating that melanoma cell-derived exosomes can promote angiogenesis in endothelial cells through the transfer of miR-424-5p.

The miR-424 has been implicated in various aspects of tumor biology, including angiogenesis 34. In nasopharyngeal carcinoma, miR-424-5p (miR-424 mature miRNA) has been shown to facilitate tumor growth, migration, and angiogenesis by targeting large tumor suppressor kinase 1 (LATS1), a key regulator of the Hippo signaling pathway [21]. Furthermore, miR-424-5p can enhance the angiogenic capacity of endothelial cells by targeting Cullin 2 (CUL2), a critical component of the E3 ubiquitin ligase complex [16]. In line with these findings, our study demonstrated that exosomal miR-424-5p derived from melanoma cells promoted the proliferation, migration, and angiogenic potential of HUVECs, contributing to tumor progression. This suggests an autocrine system developed by melanoma to support its malignant progression.

LATS2 has been characterized as a tumor suppressor gene in regulating various cellular processes, including proliferation, apoptosis, and angiogenesis [10]. In melanoma, LATS2 has been shown to inhibit cell growth and metastasis, and its downregulation is associated with poor prognosis [9]. Moreover, LATS2 can suppress angiogenesis by inhibiting the transcriptional activity of the Yes-associated protein (YAP), a key effector of the Hippo signaling pathway [28]. Consistently, in breast cancer, LATS2 has been found to inhibit tumor growth and metastasis by negatively regulating the YAP/TAZ pathway [29]. Furthermore, LATS2 has been shown to regulate endothelial cell functions, such as proliferation, migration, and tube formation, suggesting its role in modulating angiogenesis [30]. Consistent with these findings, our study identified LATS2 as a direct target of miR-424-5p, and its silencing reversed the inhibitory effects of miR-424-5p inhibition on HUVEC functions, suggesting that the exosomal miR-424-5p/LATS2 axis contributes to melanoma progression by promoting angiogenesis.

While providing valuable insights, this study is limited by its focus on experimental models, necessitating validation in clinical settings. Additionally, the specific mechanisms by which exosomal miR-424-5p regulates LATS2 and downstream angiogenic pathways require further investigation. Future research should explore the potential of exosomal miR-424-5p as a diagnostic/prognostic biomarker and therapeutic target for melanoma. Furthermore, elucidating the interplay between exosomal miR-424-5p and other miRNAs or signaling pathways involved in melanoma progression could lead to more effective treatment strategies.

## Conclusion

This study unveils melanoma cell-derived exosomal miR-424-5p as a key promoter of angiogenesis, facilitating tumor progression. Exosomal miR-424-5p enhanced endothelial cell proliferation, migration, and angiogenic potential by targeting LATS2. *In vivo*, experiments validated exosomal miR-424-5p’s ability to promote tumor growth and angiogenesis. Targeting the exosomal miR-424-5p/LATS2 axis emerges as a potential therapeutic strategy for melanoma. Future studies should validate these findings clinically, elucidate detailed mechanisms, and explore exosomal miR-424-5p’s potential as a diagnostic/prognostic biomarker and therapeutic target.

## Data Availability

The datasets used and/or analyzed during the current study are available from the corresponding author via email request.
